# Epigenetic regulation of the *ELOVL6* gene is associated with a major QTL effect on fatty acid composition in pigs

**DOI:** 10.1186/s12711-015-0111-y

**Published:** 2015-03-25

**Authors:** Jordi Corominas, Jorge AP Marchesi, Anna Puig-Oliveras, Manuel Revilla, Jordi Estellé, Estefânia Alves, Josep M Folch, Maria Ballester

**Affiliations:** Plant and Animal Genomics, Centre de Recerca en Agrigenòmica (Consorci CSIC-IRTA-UAB-UB), Edifici CRAG, Campus UAB, Bellaterra, Barcelona 08193 Spain; Departament de Ciència Animal i dels Aliments, Facultat de Veterinària, Campus UAB, Bellaterra, Barcelona 08193 Spain; INRA, UMR 1313, Génétique Animale et Biologie Intégrative, Jouy-en-Josas F, 78352 France; AgroParisTech, UMR 1313 Génétique Animale et Biologie Intégrative, Jouy-en-Josas F, 78352 France; CEA, DSV/iRCM/SREIT/LREG, Jouy-en-Josas F, 78352 France; Departamento de Mejora Genética Animal, INIA, Ctra. de la Coruña km. 7, Madrid, 28040 Spain

## Abstract

**Background:**

In previous studies on an Iberian x Landrace cross, we have provided evidence that supported the porcine *ELOVL6* gene as the major causative gene of the QTL on pig chromosome 8 for palmitic and palmitoleic acid contents in muscle and backfat. The single nucleotide polymorphism (SNP) *ELOVL6:c.-533C > T* located in the promoter region of *ELOVL6* was found to be highly associated with *ELOVL6* expression and, accordingly, with the percentages of palmitic and palmitoleic acids in *longissimus dorsi* and adipose tissue. The main goal of the current work was to further study the role of *ELOVL6* on these traits by analyzing the regulation of the expression of *ELOVL6* and the implication of *ELOVL6* polymorphisms on meat quality traits in pigs.

**Results:**

High-throughput sequencing of BAC clones that contain the porcine *ELOVL6* gene coupled to RNAseq data re-analysis showed that two isoforms of this gene are expressed in liver and adipose tissue and that they differ in number of exons and 3’UTR length. Although several SNPs in the 3’UTR of *ELOVL6* were associated with palmitic and palmitoleic acid contents, this association was lower than that previously observed with SNP *ELOVL6:c.-533C > T*. This SNP is in full linkage disequilibrium with SNP *ELOVL6:c.-394G > A* that was identified in the binding site for estrogen receptor alpha (ERα). Interestingly, the *ELOVL6:c.-394G* allele is associated with an increase in methylation levels of the *ELOVL6* promoter and with a decrease of *ELOVL6* expression. Therefore, ERα is clearly a good candidate to explain the regulation of *ELOVL6* expression through dynamic epigenetic changes in the binding site of known regulators of *ELOVL6* gene, such as SREBF1 and SP1.

**Conclusions:**

Our results strongly suggest the *ELOVL6:c.-394G > A* polymorphism as the causal mutation for the QTL on pig chromosome 8 that affects fatty acid composition in pigs.

**Electronic supplementary material:**

The online version of this article (doi:10.1186/s12711-015-0111-y) contains supplementary material, which is available to authorized users.

## Background

Elongation of very long-chain fatty acids proteins (ELOVL) are a family of enzymes that catalyze the initial and rate-limiting condensation reaction of fatty acid elongation cycle in mammals [1-3]. To date, seven ELOVL proteins have been identified i.e. ELOVL1, ELOVL3, ELOVL6 and ELOVL7 that act preferentially on saturated fatty acids (SFA) and monounsaturated fatty acids (MUFA) and ELOVL2, ELOVL4 and ELOVL5 that act preferentially on polyunsaturated fatty acids (PUFA) [4-6]. In mammals, the enzyme ELOVL6 catalyzes the elongation of long-chain SFA and MUFA with 12 to 18 carbon atoms and is considered as a key gene in the control of the overall balance of fatty acid composition [2,7]. Expression of the gene coding for ELOVL6 is highly up-regulated, both in liver and adipose tissue in the refed state compared to fasting state, which indicates that this enzyme has a major role in the synthesis of long-chain fatty acids [8].The porcine *ELOVL6* gene is located on chromosome 8 (SSC8, SSC for *Sus scrofa*), in a region where a quantitative trait locus (QTL) that affects palmitic and palmitoleic acid contents was previously detected [2,9]. Moreover, it was recently reported that this gene is differentially expressed in adipose tissue from Iberian x Landrace backcross animals with extreme phenotypic differences in intramuscular fatty acid composition [10].

Expression of *ELOVL6* was first identified in the liver of transgenic mice that over-expressed sterol regulatory element binding transcription factors (SREBF) [1]. SREBF are transcription factors that control the expression of genes involved in *de novo* lipogenesis [11]. In tissues that synthesize fatty acids *de novo*, expression of SREBF is highly correlated with that of key lipogenic genes involved in this metabolic pathway [12]. Transcriptional regulation of *ELOVL6* by SREBF was also confirmed by using DNA microarrays to analyze the expression of *ELOVL6* in transgenic mice overexpressing *SREBF1*. [13], and by analyzing the promoter region of mouse *ELOVL6* [14]. Kumadaki et al. [14] demonstrated that in mouse liver, nuclear SREBF1 activates the *ELOVL6* promoter by interacting with two sterol response elements (SRE). However, although SREBF1 can bind to E-box motifs, there was no evidence that E-box motifs were involved in *ELOVL6* activity [14,15]. Results of our previous analysis on the promoter of pig *ELOVL6* [2] showed that: (1) pig and mouse *ELOVL6* promoters share SRE and E-box motifs, and in the pig *ELOVL6* promoter, SRE elements are present at positions −18, −450 and −524 and an E-box motif at position −331; (2) a single nucleotide polymorphism (SNP) i.e. *ELOVL6:c.-533C > T* is located close to the most distal SRE element and is highly associated with percentages of palmitic and palmitoleic acids in muscle and backfat and with the expression level of *ELOVL6* in backfat; (3) the pig *ELOVL6* promoter contains binding sites for other transcription factors i.e. for SP1 transcription factor (SP1) at position −470 with a SNP at position −480 i.e. *ELOVL6:c.-480C > T* and for MLX interacting protein-like (MLXIPL) at position −322 (also called carbohydrate response element binding protein or ChREBP); (4) the pig *ELOVL6* promoter contains five additional SNPs (*ELOVL6:c.-574C > T, ELOVL6:c.-534C > T*, *ELOVL6:c.-492G > A, ELOVL6:c.-394G > A* and *ELOVL6:c.-313C > T*); and (5) expression of *ELOVL6* varies between various lipogenic tissues (liver, adipose tissue and muscle), which suggests that the mechanisms that regulate the expression of this gene differ in each tissue. In addition, we performed a whole-genome association study of the expression levels of *ELOVL6* (eGWAS) in liver, adipose tissue and muscle and identified several genomic regions that may be involved in the tissue-specific expression of this gene [2]. Epigenetic modifications is another mechanism that can contribute to these tissue-specific differences in the expression of *ELOVL6* [16]. DNA methylation is one of the major epigenetic mechanisms that regulates gene transcription and it was shown to be involved in the regulation of genes associated to lipid metabolism, such as *fatty acid desaturase 2* (*FADS2*) [17] or *peroxisomal proliferator-activated receptor alpha* (*PPARα*) [18]. Finally, one cannot exclude the possibility that microRNAs, a class of short non-coding RNAs with a key role in gene expression, may affect expression of *ELOVL6*, since the 3’UTR of porcine *ELOVL6* gene has not been fully characterized.

The overall objective of the present study was to investigate the mechanisms that contribute to the control and regulation of *ELOVL6* expression and their influence on porcine meat quality traits. Thus, we characterized the 3’UTR of porcine *ELOVL6* and identified several polymorphisms. In addition, we performed a methylation study of the *ELOVL6* promoter region on DNA extracted from liver, adipose tissue, muscle and spleen, to determine whether epigenetic modifications play a role in the differential expression of *ELOVL6* across tissues.

## Methods

### Animals

The population analyzed was generated by crossing three Iberian (Guadyerbas line) boars with 31 Landrace sows (the so-called IBMAP cross) [19], and contained several generations and backcrosses. The animals used in this study belonged to the backcross (BC1_LD) that was produced by crossing five F1 (Iberian x Landrace) boars with 26 Landrace sows and resulted in 144 backcrossed animals. All pigs were raised and fed under the standard intensive system in Europe and feeding was *ad libitum* with a cereal-based commercial diet. Pigs were slaughtered at an average age of 179.8 ± 2.6 days following national and institutional guidelines for the Good Experimental Practices and approved by the Ethical Committee of the Institution (IRTA- Institut de Recerca i Tecnologia Agroalimentàries). Samples of liver, muscle (*longissimus dorsi*) and adipose tissue (backfat) were collected, snap-frozen in liquid nitrogen and stored at −80°C. Genomic DNA was obtained from blood and liver samples from the 144 animals according to the phenol-chloroform method, as described elsewhere. Backfat [20] and intramuscular fatty acid composition [9] was measured with a protocol based on gas chromatography of methyl esters [21].

### BAC screening and sequencing

The porcine bacterial artificial chromosome (BAC) INRA library (Centre de Ressources Biologiques Génomique des Animaux Domestiques et d’Intérêt Economique i.e. CRB-GADIE; http://crb-gadie.inra.fr) was used to select BAC clones containing the SSC8 region that carries *ELOVL6*. This BAC library constructed with the pBeloBAC11 vector comprises 107 520 clones with an average insert size of 135 kb, representing a five-fold coverage of the pig haploid genome [22]. Screening of the library was performed using three sets of primers located respectively in the promoter region, second exon (intermediate gene region) and at the end of the coding region in exon 4, in order to encompass the major part of *ELOVL6* gene (See Additional file 1: Table S1). Primers were designed using the software PRIMER3 [23] and validated using the software PRIMER EXPRESS^TM^ (Applied Biosystems). BAC screening was performed by two-step PCR according to CRB-GADIE protocols (PCR of superpools and pools) and positive BAC clones were confirmed by checking the size of their PCR-amplified fragments.

BAC clones were cultured on Luria-Bertani (LB) agar containing 12.5 μg/mL of chloramphenicol overnight at 37 °C and then, isolated clones were grown in 4 ml LB broth with 12.5 μg/mL of chloramphenicol overnight at 37 °C in a shaking incubator. Finally, 4 mL of the overnight starter culture was inoculated in 500 ml LB-medium supplemented with chloramphenicol (12.5 μg/mL) and incubated in the same conditions to an optical density of 2. BAC DNA was isolated using the plasmid DNA purification Nucleobond BAC100 kit (Macherey-Nagel), following the manufacturer’s recommendations of the low-copy plasmid purification (Maxi BAC100) section. DNA was quantified using the Nano-Drop ND-1000 spectrophotometer (NanoDrop products) and checked for purity and integrity by electrophoresis on agarose gels.

For each BAC, bar-coded libraries were generated using the Ion Xpress Plus fragment library kit (Life Technologies) with an insert size of approximately 250 bp. Libraries were sequenced on a Personal Genome Machine (PGM) Ion Torrent instrument (Life Technologies) using an Ion 314R chip. More than 220 000 single-end reads were generated with an average read length of 152 bp for each library.

### *De novo* assembly and characterization of the porcine *ELOVL6* gene

Statistics of reads and quality control were determined with FASTQC [http://www.bioinformatics.babraham.ac.uk/projects/fastqc/]. All reads were mapped against the *Escherichia coli* genome using the Burrows-Wheeler Alignment tool (bwa v.0.6.2) [24], in order to discard all reads corresponding to bacterial DNA. Removal of sequence adapters, quality trimming of reads and *de novo* assembly were performed using the *de novo* assembler tool of CLC Genomics Workbench v.6.0.1 [http://www.clcbio.com]. The 3’UTR sequence of *ELOVL6* was identified from *de novo* assembled reads aligned with the corresponding human (GenBank:NM_001130721) and bovine (GenBank:NM_001102155) genes, using the Basic Local Alignment Search Tool (BLAST v2.2.28) [25]. The resulting pig *ELOVL6* 3’UTR sequence was used as reference sequence to align reads from liver (12 BC1_LD animals) and adipose tissue (6 BC1_LD animal) transcriptomes that were obtained in previous studies [10,26] by using the software TopHat v2.0.1 [27,28] and including the pig genome (*Sscrofa10.2*) [http://www.ensembl.org/info/data/ftp/index.html] as a combined reference. Finally, SNPs in the *ELOVL6* gene were manually identified by comparing the reference sequence with mapped reads with the Integrative Genomic Viewer (IGV v.2.1) [29,30].

### Genotyping

SNPs *ELOVL6:c.1408C > T* and *ELOVL6:c.1922C > T* were genotyped using the platform KASP SNP genotyping system [http://www.lgcgroup.com/products/kasp-genotyping-chemistry/#.VQAP4fmG9ak]. A total of 179 animals including the 144 BC1_LD backcross animals and their corresponding 35 parents from the IBMAP cross (F_0_ and F_1_) were genotyped.

For the genome-wide association study (GWAS), the 144 animals of the BC1_LD backcross were genotyped with the Porcine SNP60 Illumina BeadChip using the Infinium HD Assay Ultra protocol (Illumina). Raw data had a high genotyping quality (call rate > 0.99) and were visualized and analyzed with the Illumina GenomeStudio software. For subsequent data analysis, a subset of 54 998 SNPs was selected by removing SNPs with a minor allele frequency less than 5%, SNPs with more than 5% missing genotype data and SNPs that were duplicated in the *Sscrofa10.2* assembly.

### Chromosome-wide association analyses

Association analyses of whole-genome SNP genotypes, together with the previously identified SNPs *ELOVL6:c.-533C > T*, *ELOVL6:c.-480C > T*, *ELOVL6:c.416C > T* [2] and the newly detected *ELOVL6:c.1408A > G* and *ELOVL6:c.1922C > T* SNPs were performed on the following phenotypes: RT-qPCR expression data of *ELOVL6* mRNA in backfat and C16:0 and C16:1(n-7) fatty acids composition in backfat and intramuscular fat. The position of the SNPs was based on the *Sscrofa*10.2 genome assembly [http://www.animalgenome.org/repository/pig/]. GWAS were performed with a mixed model [31,32] that accounted for additive effects associated with each marker (see below) by using the Qxpak 5.0 software [33]:

$$ {\mathrm{y}}_{\mathrm{i}\mathrm{jlkm}} = \mathrm{S}\mathrm{e}{\mathrm{x}}_{\mathrm{i}} + \mathrm{Batc}{\mathrm{h}}_{\mathrm{j}}+{\uplambda}_{\mathrm{l}}{\mathrm{a}}_{\mathrm{k}} + {\mathrm{u}}_{\mathrm{l}} + {\mathrm{e}}_{\mathrm{i}\mathrm{jlkm}}, $$

in which y_ijlkm_ is the l-th individual’s record, sex (two levels) and batch (five levels) are fixed effects, λ_l_ is a −1, 0, +1 indicator variable depending on the l-th individual’s genotype for the k-th SNP, a_k_ represents the additive effect associated with the k-th SNP, u_l_ represents the polygenic effect for individual l, treated as random and distributed as N(0, **A**σ_u_), where **A** is the numerator of the kinship matrix, and e_ijlkm_ is the residual. The polygenic effect allows us to account for family relationships. In this analysis, each SNP was tested individually for association with phenotype. The R package q-value [34] was used to calculate the FDR-based q-value to measure the statistical significance at the genome-wide level for association studies. The cut-off for a significant association at the chromosome level was set at a q-value ≤ 0.05. In addition, carcass weight (βc_l_) was added to the model as a covariate for fatty acid composition:

$$ {\mathrm{y}}_{\mathrm{i}\mathrm{jlkm}} = \mathrm{S}\mathrm{e}{\mathrm{x}}_{\mathrm{i}} + \mathrm{Batc}{\mathrm{h}}_{\mathrm{j}}+\upbeta {\mathrm{c}}_{\mathrm{l}}+{\uplambda}_{\mathrm{l}}{\mathrm{a}}_{\mathrm{k}} + {\mathrm{u}}_{\mathrm{l}} + {\mathrm{e}}_{\mathrm{i}\mathrm{jlkm}}. $$

The same model was used to determine the effect of haplotypes on the traits of interest. The only difference is that haplotypes were treated as random additive effects, in contrast to the individual SNPs, which were considered as fixed additive effects.

### DNA methylation analyses

DNA methylation analyses of liver and backfat were performed on 43 animals, while muscle and spleen that were chosen as control tissues expressing low levels of *ELOVL6* were analyzed on six animals. DNA was extracted using the phenol-chloroform method, as described elsewhere. Methylation studies were performed using the bisulfite methodology [35] and the pyrosequencing technique [36,37]. The bisulfite gDNA conversion was performed on 500 ng of genomic DNA from each sample with the EZ DNA Methylation kit (Zymo Research). The regions of interest were amplified using primers (See Additional file 1: Table S1) that were designed from the resulting methylated sequence using the allele quantification assay type of the PSQ assay design software (Biotage). PCR were performed in 25 μL samples containing 0.6 units of AmpliTaq Gold (Applied Biosystems), 1.5 to 2.5 mM MgCl_2_ (depending on the primers; (See Additional file 1: Table S1)), 0.2 mM of each dNTP, 0.5 μM of each primer and 25 ng of treated genomic DNA. PCR were carried out under the following conditions: 94°C for 10 min, 40 cycles of 94°C for 1 min, 60°C for 1 min and 72°C for 1 min, with a final extension at 72°C for 7 min. Pyrosequencing analysis was carried out on a PSQ HS 96A system with the Pyro Gold sequence analysis (SQA) reagent (Biotage) using specific pyrosequencing primers for each region (See Additional file 1: Table S1). Statistical comparison of methylation values between tissues, genotypes and gene expression data was made using an ANOVA test in R considering sex and batch. RNA extraction, cDNA synthesis and gene expression analysis by real time quantitative PCR (RT-qPCR) were performed following the procedure described in [2].

## Results and discussion

### Pig *ELOVL6* gene structure and identification of a new isoform

**Figure 1:**
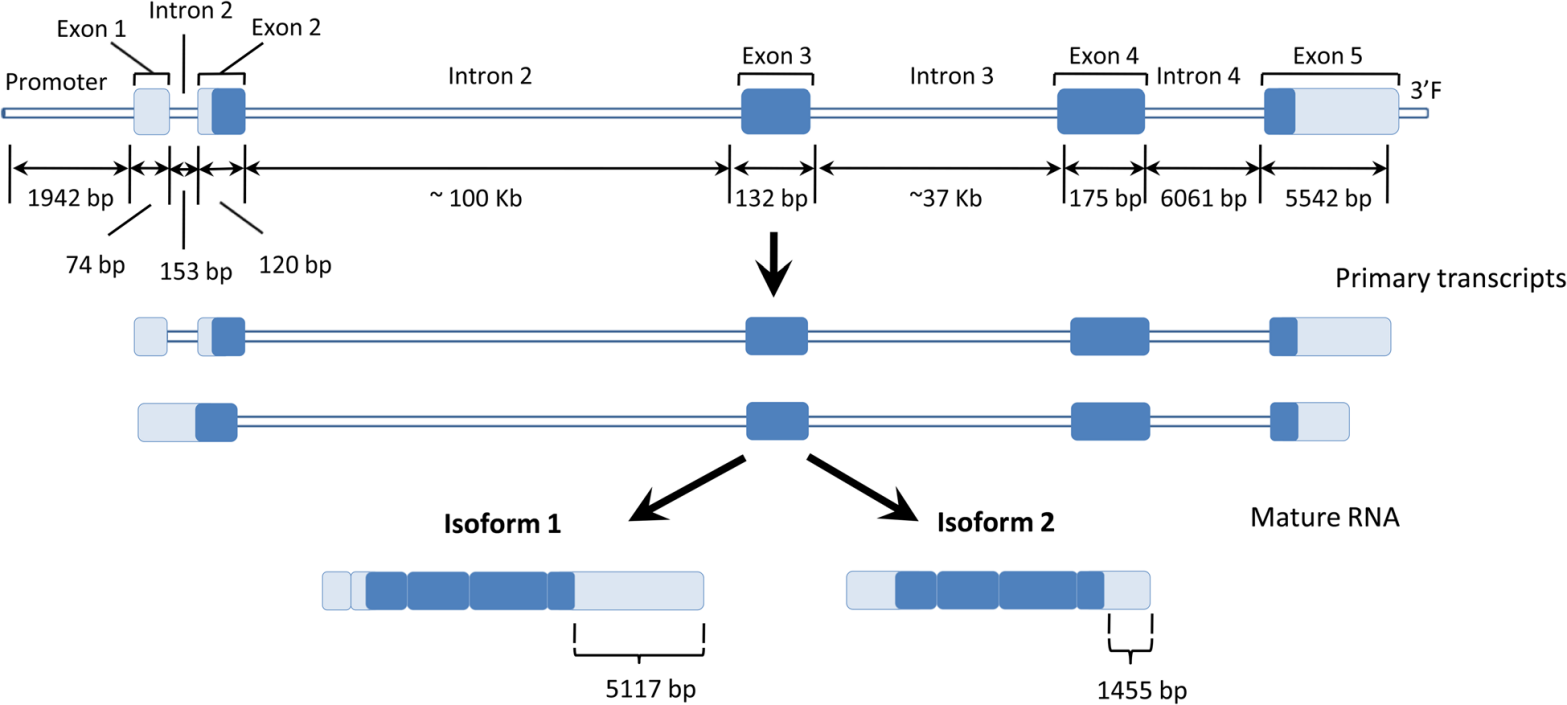
**Genetic architecture of the porcine** ***ELOVL6*** **gene, with the two transcribed variants identified by BAC sequencing and RNAseq analysis.** Exons are indicated by blue boxes with dark blue corresponding to sequences that code for ELOVL6 protein and pale blue to 5’UTR and 3’UTR regions.

Despite the important role played by *ELOVL6* in lipid metabolism [2,3,7], a comparison of human, bovine and porcine *ELOVL6* mRNA sequences revealed that the current sequence of the porcine *ELOVL6* gene is incomplete (data not shown). In order to characterize pig *ELOVL6*, we screened a pig BAC library for its promoter region, exon 2 (intermediate region) and exon 4 (terminal region). Six BAC clones that contain at least one of these three regions were identified by PCR: BAC 651E12, 650D01 and 385A04 were positive for the promoter region, BAC 201D05, 95C02, 754E02 and 385A04 were positive for exon 2 and BAC 754E02 was positive for exon 4. Of these six BAC clones, only BAC 385A04 and 754E02 were positive for two different sequences, i.e. BAC 385A04 contained the promoter and exon 2 regions and BAC 754E02 contained exons 2 and 4, which suggests that these two clones cover most of the porcine *ELOVL6* gene. DNA of both BAC clones was sequenced with the PGM of Ion Torrent and around 265 000 single-end reads were generated for each BAC with an average read length of 152 bp. The data generated was used to perform a *de novo* assembly of porcine *ELOVL6* with the CLC Genomics Workbench v.6.0.1. The 129 672 bp long sequence contained 1942 bp of the upstream region, four introns and five exons of porcine *ELOVL6* (Figure 1). The protein coding region starts at position 2201 bp and ends at position 123 132 bp of this sequence (Figure 1). To validate the new gene annotation, RNA-Seq data from adipose tissue and liver transcriptomes [10,26] were used to map the reads against the new *ELOVL6* sequence. The alignments obtained were concordant with the proposed *ELOVL6* gene structure, but some reads were located between the first and second exons. In addition, the number of mapped reads was clearly reduced in the middle of the fifth exon. Interestingly, a poly-A signal was identified in this region (at position 124 578 bp), which indicates the end of an alternative isoform (Figure 1). Therefore, as for the human *ELOVL6* gene, two different isoforms of porcine *ELOVL6* are expressed in both liver and adipose tissue. These isoforms differ in: (i) total number of exons, i.e. four in variant 2 (the first and second exons are combined into a single exon) against five in variant 1, and (ii) length of the 3’UTR, which is shorter in variant 2 (1455 bp) than in variant 1 (5117 bp) (Figure 1).

### Identification of polymorphisms in the 3’UTR of porcine *ELOVL6*

Alignment and analysis of all mapped reads from RNA-Seq data [10,26] allowed us to identify 11 SNPs (Table 1) in the 3’UTR of porcine *ELOVL6*, among which five were found in both variants and six were found only in variant 2. All SNPs were arranged in three haplotypes, which can be distinguished by genotyping *ELOVL6:c.1408A > G* and *ELOVL6:c.1922A > G* SNPs. Hence, these two tag SNPs were genotyped in IBMAP founders, parental BC1_LD animals and the BC1_LD population. Regarding the IBMAP founders, alleles *ELOVL6:c.1408G* and *ELOVL6:c.1922G* were fixed in Iberian boars. The *ELOVL6:c.1408A* allele was fixed in the founder Landrace sows, whereas the allele *ELOVL6:c.1922A* had a frequency of 0.7 in these sows. In BC1_LD Landrace sows, allelic frequencies of *ELOVL6:c.1408A* and *ELOVL6:c.1922A* were 0.94 and 0.38, respectively. Both *ELOVL6:c.1408A > G* and *ELOVL6:c.1922A > G* SNPs segregated in the BC1_LD animals with frequencies for allele A equal to 0.72 and 0.46, respectively.

**Table 1:** **Polymorphisms identified in the 3’UTR of the** ***ELOVL6*** **gene**

**Position (bp)** ^**1**^	**Polymorphism**	**Isoform**
1408^2^	A/G	Variant 1 and 2
1817	C/T	
1922^2^	A/G	
2070	C/G	
2532	A/G	
3599	G/T	Variant 2
3834	A/G	
4750	A/G	
4765	G/T	
4967	A/C	
5233	A/C	

^1^Positions relative to the transcription start site, TSS, of the GenBank: NW_003610943; ^2^SNPs genotyped in the BC1_LD population.

It is well known that the presence of polymorphisms in the 3’UTR of genes may affect the binding of microRNAs. This interaction is important for the regulation of gene expression, since microRNAs mediate translational repression and mRNA destabilization [38]. For instance, microRNA miR-33a/b has been described as a potential regulator of lipid metabolism by repressing the translation of genes coding for key enzymes that are involved in cholesterol efflux (*ABCA1* and *NPC1*), fatty acid metabolism (*CROT* and *CPT1a*) and insulin signaling (*IRS2*) [39,40]. To assess if the polymorphisms present in the 3’UTR of *ELOVL6* affect the disruption or creation of microRNA binding sites, a computer-assisted identification of potential microRNA binding elements was performed using the finder tool of patrocles programme [41]. Twelve putative microRNA binding sites were found to be modified by the 11 detected SNPs. MicroRNAs miR-524-3p, miR-525-3p, miR-18a/b, miR-204 and miR-211 were predicted to bind to both mRNA isoforms, whereas miR-584, miR-452, miR-603, miR-1262, miR-490-5p, miR-30a/d/e and miR-335 were predicted to bind only to variant 2. These predictions suggest that microRNAs may be involved in the regulation of porcine *ELOVL6*. However, further studies are needed to elucidate their role in the differential expression of *ELOVL6* in pig adipose tissue, liver and muscle.

### Association studies support the major role of the *ELOVL6:c.-533C > T* polymorphism

**Figure 2:**
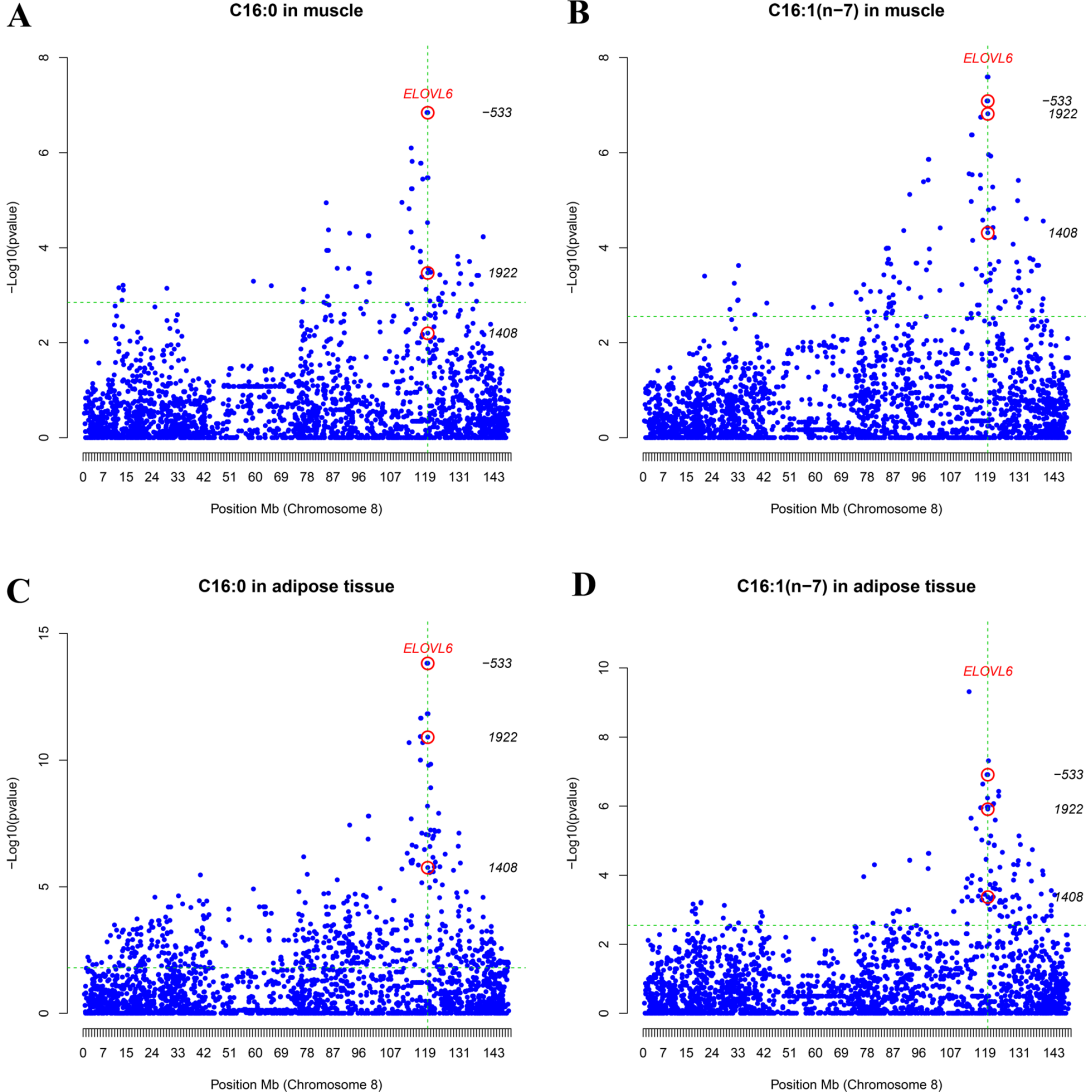
**Association analysis between SNP genotypes for SSC8 and the percentages of: palmitic (A) and palmitoleic (B) acid in muscle and palmitic (C) and palmitoleic (D) acid in backfat.** *ELOVL6* polymorphisms are included and labeled with a red circle. Positions in Mb are relative to the *Sscrofa 10.2 assembly* of the pig genome. The vertical dashed line indicates the position of *ELOVL6* gene and the horizontal dashed line marks the chromosome-wide significance level (FDR-based q-value ≤ 0.05).

Previously, we found that SNP *ELOVL6:c.-533C > T* in the promoter region of *ELOVL6* was associated with a QTL on SSC8 that affects palmitic and palmitoleic acid contents in muscle and backfat [2]. This SNP explained a large part of the phenotypic variance of each of these traits in both BF and IMF tissues: 18% for C16_IMF_, 32% for C16_BF_, 20% for C16:1(n-7)_IMF_ and 19% for C16:1(n-7)_GD_. Nevertheless, we cannot exclude that other SNPs may also have a major association with these traits. The two newly genotyped 3’UTR SNPs and the three SNPs previously described in [2] were added to the 2565 SNPs on SSC8 that are included in the Porcine Illumina SNP60 BeadChip, in order to perform association analyses on 136 BC1_LD animals for FA composition in muscle and backfat. In this analysis, SNP *ELOVL6:c.1922A > G* showed a significant association with the percentages of palmitic acid in muscle (p-value = 3.38×10^−04^) and backfat (p-value = 1.23x10^−11^) (Figure 2A and C). In contrast, SNP *ELOVL6:c.1408A > G* showed significant associations only with percentage of palmitic acid in backfat (p-value = 1.73x10^−06^) (Figure 2C). In addition, percentage of palmitoleic acid was significantly associated with SNP *ELOVL6:c.1922A > G* in both muscle (p-value = 1.51×10^−07^) and backfat (p-value = 1.22x10^−06^) (Figure 2B and D). Significant associations were also found between palmitoleic acid and SNP *ELOVL6:c.1408A > G* in muscle (p-value = 4.86×10^−05^) and backfat (p-value = 4.24×10^−04^) (Figure 2B and D). The effect of the haplotypes formed by the combination of these two SNPs (*ELOVL6:c.1408A > G* and *ELOVL6:c.1922A > G*) on the different traits was tested, but no significant associations were observed (data not shown). Nevertheless, for both FA, SNP *ELOVL6:c.-533C > T* always showed a greater association than the 3’UTR SNPs (Figure 2), which further supports its role in the determination of the SSC8 QTL. No significant associations were observed between the SNPs in the 3’UTR sequence and *ELOVL6* expression levels in backfat, liver and muscle (data not shown).

Taken together, these results indicate that SNP *ELOVL6:c.-533C > T* in the promoter of *ELOVL6* is the most promising candidate among the genotyped SNPs on SSC8. However, to further support the main role of this SNP, an association study was performed to compare the individual effect of SNPs *ELOVL6:c.-533C > T* and *ELOVL6:c.1922A > G* against the effect of the haplotypes formed by the combination of these two SNPs. These analyses were performed with a reduced number of animals (n = 94), for which the allele origin (Iberian or Landrace) could be unambiguously determined based on pedigree information. As expected, the effect of SNP *ELOVL6:c.-533C > T* was greater than that of the haplotypes for all analyzed traits (p-value _backfat gene expression_ = 3.68×10^−03^, p-value _IMF_C16:0_ = 1.33×10^−03^, p-value _IMF_C16:1(n-7)_ = 3.72×10^−04^, p-value _BF_C16:0_ = 6.15×10^−10^ and p-value _BF_C16:1(n-7)_ = 9.15×10^−04^) (See Additional file 2: Table S2). In conclusion, our results confirm that SNP *ELOVL6:c.-533C > T* plays a key role in explaining the SSC8 QTL that affects palmitic and palmitoleic acid contents in pig. However, we cannot exclude the possibility that the 3’UTR may have a secondary role on the regulation of *ELOVL6* expression or that other variants located in intronic regions may be involved.

### Promoter methylation is an additional level in the regulation of porcine *ELOVL6* expression

**Figure 3:**
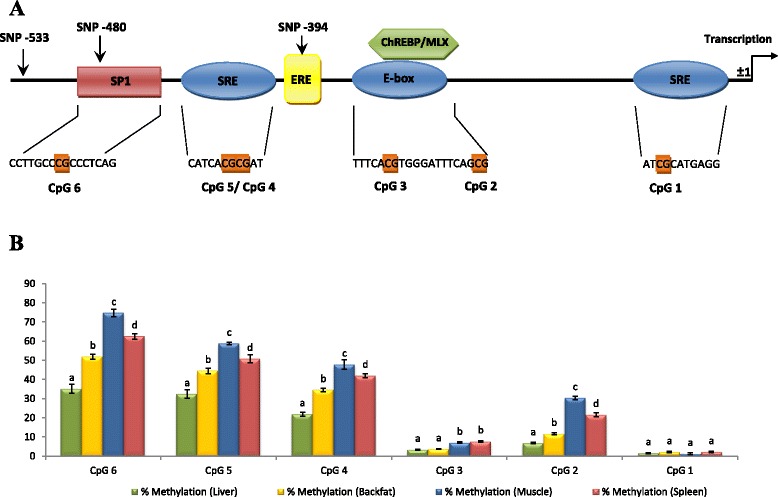
**Characterization of the methylation patterns on the** ***ELOVL6*** **gene promoter. (A)** Schematic representation of the transcription factor binding elements studied, together with the CpG-sites analyzed in the methylation study. **(B)** Plot showing the percentages of methylation observed for each CpG-site in four porcine tissues: liver, backfat, muscle and spleen. Data represent means ± SEM. Values with different superscript letters (a, b, c and d) indicate significant differences between groups (p-value < 0.05), as determined by a linear model in R.

**Figure 4:**
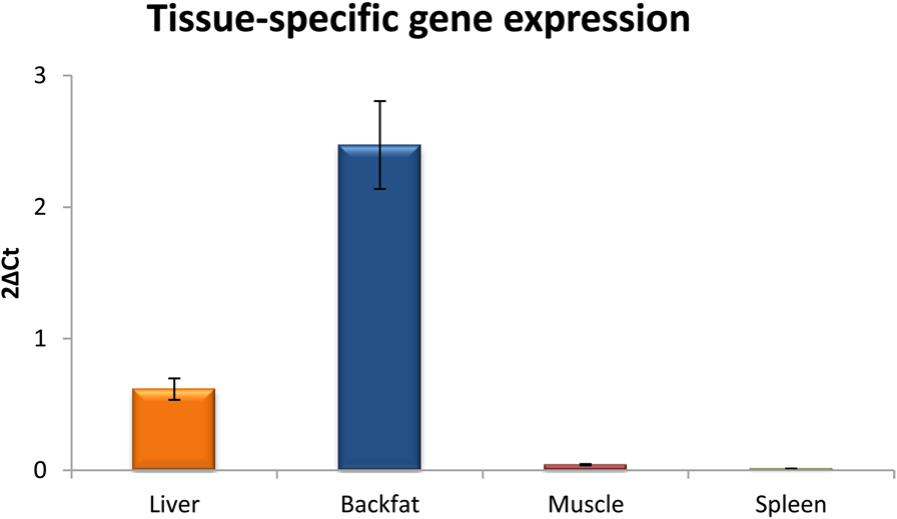
**Tissue-specific differences in** ***ELOVL6*** **gene expression among liver, adipose tissue, muscle and spleen.** Gene expression quantification was performed by real-time quantitative PCR (RT-qPCR), according to the procedure described in [2]. Gene expression was compared using the 2ΔCt data obtained from quantitative PCR analyses. Data represent mean ± SEM.

DNA bisulfite conversion was used to compare the methylation patterns of the promoter region of pig *ELOVL6* between liver, adipose tissue (backfat), muscle and spleen (tissues with low levels of *ELOVL6* expression). The methylation study was focused on CpG-sites whose methylated states may affect the binding of SREBF1, the most relevant transcription factor of *ELOVL6*. All individual CpG-sites identified in the SRE and E-box motifs of *ELOVL6* promoter [2] were included in the study. In addition, it was observed that SP1 is required as an additional regulator for SREBF1 activity in several lipogenic genes [1,42]. Interestingly, a CpG-site was identified in the SP1 binding element, in which SNP *ELOVL6:c-416C > T* [2] is located (Figure 3A), making this CpG a clear candidate. Finally, six CpG sites, that covered the major part of the described promoter, were analyzed (Figure 3A). Methylation analysis of these CpG sites showed that methylation levels were higher in muscle and spleen than in liver and adipose tissue (Figure 3B). These results are in agreement with the lower level of *ELOVL6* expression observed in the former tissues (Figure 4), which suggests that an epigenetic mechanism may be involved in the regulation of *ELOVL6* mRNA abundance.

The six selected CpG sites were distributed in two clear regions with opposite levels of methylation i.e. (i) lower methylation levels in the proximal region (−349 bp to −1 bp) and (ii) higher methylation levels in the distal region (−529 bp to -350 bp) (Figure 3B). On the one hand, the low methylation levels in the proximal region (CpG1, CpG2 and CpG3) in all tissues, suggest that this region may be important for maintaining a basal gene expression. On the other hand, the higher methylation levels in the distal promoter regions (CpG4, CpG5 and CpG6), suggest that methylation of these motifs may be relevant for the regulation of *ELOVL6* expression among tissues. Statistical analyses showed significant lower methylation levels of these sites in liver than in backfat (p-value CpG4 = 7.27×10^−09^, p-value CpG5 = 1.18×10^−05^ and p-value CpG6 = 1.04×10^−07^), muscle (p-value CpG4 = 2.13×10^−14^, p-value CpG5 = 1.52×10^−10^ and p-value CpG6 = 1.82×10^−14^) and spleen (p-value CpG4 = 4.42×10^−11^, p-value CpG5 = 5.24×10^−07^ and p-value CpG6 = 1.47x10^−10^) (Figure 3B). Methylation levels in adipose tissue were clearly higher than in liver, although expression of *ELOVL6* is higher in adipose tissue than in liver [2]. The higher levels of *ELOVL6* expression in adipose tissue are explained by the major role of this tissue in lipogenic pathways in pigs [43,44] and, consequently, lipogenic genes are upregulated by SREBF1 in this tissue [43]*.* In agreement, gene expression correlation analysis performed in our animal material, showed a high correlation between *SREBF1* and *ELOVL6* (r = 0.77) in adipose tissue, but not in liver (Ballester *et al.*, unpublished). Thus, these results allowed us to hypothesize that methylation of the *ELOVL6* promoter region between −529 bp and −350 bp may be one possible mechanism responsible for the differential expression of this gene among the tissues analyzed. This region contains one methylated SRE element and one SP1 binding site, which are well-known transcription factors involved in the regulation of lipogenic genes such as *ELOVL6* [1,2,14,42]. Nevertheless, since several SRE elements have been identified in the promoter region of *ELOVL6*, a site-specific chromatin immunoprecipitation (ChIP) approach is required to investigate the capacity of SREBF1 to bind the specific methylated SRE [45,46], as well as the effect of the methylation level on SREBF1 binding. Regarding SP1 elements, some studies have reported that variations in the SP1 binding site can reduce the methylation level of CpG sites [47,48]. In our case, we found that the SP1 element contains SNP *ELOVL6:c-416C > T* that was reported in [2], which suggests a putative protective role against methylation. In our data, this putative protective role was also observed in the methylation levels of CpG5 (p-value = 1.13×10^−03^) and CpG4 motifs (p-value = 5.43×10^−04^) and a suggestive significant effect in the CpG6 motif (p-value = 8×10^−02^) in adipose tissue. Nevertheless, functional studies are needed to investigate the binding of SP1 to the *ELOVL6* promoter and its role in the control of the expression of *ELOVL6*.

Moreover, it has been shown that some transcription factors are capable of regulating dynamic methylation cycles that lead to rapid changes in the methylation levels of the promoter of the regulated gene [49]. This additional level of regulation may affect the regulation of *ELOVL6* expression in response to physiological changes.

### Role of the *ELOVL6* promoter in the regulation of its expression

**Figure 5:**
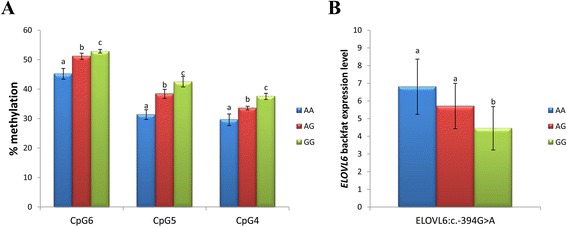
**Effect of the** ***ELOVL6:c.-394G > A*** **polymorphism on** ***ELOVL6*** **gene expression regulation. (A)** Plot showing the percentage of methylation observed for each CpG-site according to *ELOVL6:c.-394G > A* genotypes. **(B)** Plot showing *ELOVL6* expression levels in backfat according to *ELOVL6:c.-394G > A* genotypes. Data represents means ± SEM. Values with different superscript letter (a, b and c) indicate significant differences between groups (p-value < 0.05) as determined by a linear model in R.

Previous studies have shown that estrogen receptor alpha (ERα) can cause rapid epigenetic modifications that influence the regulation of gene expression [49,50]. Activation of ERα, by estradiol binding or phosphorylation of serine 118, modulates its three-dimensional surface, causing a recruitment of coactivator complexes, including DNA methyltransferases (DNMT) [49]. Hence, to assess if ERα binding may be related to the different methylation patterns detected in the promoter of *ELOVL6* and, consequently to the expression of *ELOVL6*, a computer-assisted identification of putative ERα binding sites was performed using the LASAGNA-Search software [51]. Interestingly, an estrogen response element (ERE) was predicted at position −397 to −382, between the two regions with different methylation patterns (Figure 3A). The minimal consensus ERE sequence is a 13 bp palindromic inverted repeat: 5'-GGTCAnnnTGACC-3' [52,53]. However, most estrogen-regulated genes contain imperfect ERE with variations of one or more nucleotides from the consensus sequence. It has been reported that up to two changes in the ERE consensus sequence may still allow the binding of ER with the appropriate flanking sequences adjacent to the core [52]. In this way, although the ERE sequence in the *ELOVL6* promoter presents two base changes from the consensus: 5’-GG*G*C*T*nnnTGACC-3’, the immediate flanking sequences 5’-CAGG*G*C*T*nnnTGACCTG-3’ may be sufficient to retain ERE function [52,53]. However, it should be noted that the consensus ERE sequence of the porcine *ELOVL6* promoter, contains SNP *ELOVL6:c.-394G > A*, which constitutes a third mutation in the half-site of the ERE palindrome that may prevent the ER-ERE binding [52]. Interestingly, genotyping data showed that *ELOVL6:c.-394G > A* and *ELOVL6:c.-533C > T* SNPs are in complete linkage disequilibrium. Statistical analyses showed a significant association between SNP *ELOVL6:c.-394G > A* and CpG6 (p-value = 1.29×10^−03^), CpG5 (p-value = 6.92×10^−04^) and CpG4 (p-value = 7.41×10^−04^) in adipose tissue. Animals homozygous for the *ELOVL6:c.-394G* allele showed a higher methylation rate (Figure 5A). Accordingly, a significant association was also observed between *ELOVL6* expression levels and SNP *ELOVL6:c.-394G > A* (p-value = 3.25×10^−02^) (Figure 5B), as previously described [2]. Based on RNA-Seq data [10], the ratio of differential allelic expression between *ELOVL6:c.-394G > A* alleles was found to be greater than 1.5 (A:G). Finally, a significant association was observed between *ELOVL6* expression and CpG5 (p-value = 3.47x10^−02^) and a suggestive effect was found between *ELOVL6* expression and CpG6 (p-value = 6.79×10^−02^).

Taken together, the results suggest a new mechanism for the regulation of the expression of *ELOVL6*. The hypothetical alteration of ERα binding by SNP *ELOVL6:c.-394G > A* may be the main factor responsible for the differential expression of *ELOVL6* in adipose tissue. In the methylated region that contains two SRE elements, the SRE element between positions −460 and −449 constitutes a clear candidate for methylation changes. Its core binding site contains two CpG sites (CpG4 and CpG5) (Figure 3A), whose methylation levels are associated with the *ELOVL6:c.-394G > A* genotype and *ELOVL6* expression. In addition, previous studies in mouse have shown that this SRE element has a relevant effect on *ELOVL6* expression [14]. Hence, inhibition of SREBF1 binding caused by the methylation of SRE elements in the promoter of *ELOVL6*, in particular the SRE element located between positions −460 and −449, is the most likely mechanism responsible for the reduction of *ELOVL6* expression. Previously, we showed that a reduced *ELOVL6* expression is associated with an accumulation of palmitic and palmitoleic acids in muscle and adipose tissue [2]. Thus, variation in *ELOVL6* expression can modulate fatty acid composition in muscle and backfat and have important sensorial and technological consequences on meat quality [54] and insulin sensitivity [7]. Our results provide genetic evidence to support SNP *ELOVL6:c.-394G > A* as the causal mutation of the QTL on SSC8 but additional investigations are necessary to validate its effect on ERα binding. Furthermore, to our knowledge, this is the first study that suggests a mechanism for the regulation of *ELOVL6* expression in pigs. Therefore, based on the metabolic similarities between pigs and humans [43], the regulatory mechanism described here may be useful to improve knowledge on human lipid-related diseases, such as obesity, diabetes or metabolic syndrome.

## Conclusions

In this paper, we describe the complete genetic structure of porcine *ELOVL6* gene and show that two different isoforms are expressed in both liver and adipose tissue. SNP *ELOVL6:c.-533C > T* was found to be more strongly associated with the expression of *ELOVL6* and with the percentages of palmitic and palmitoleic acids in *longissimus dorsi* and adipose tissue than the two genotyped SNPs of the 3’UTR region. These results indicate that the promoter region of *ELOVL6* may be the main regulatory region involved in the variation of *ELOVL6* expression in pigs. Interestingly, SNP *ELOVL6:c.-394G > A*, which is in linkage disequilibrium with SNP *ELOVL6:c.-533C > T* and is located in the only ERα binding site predicted in the promoter of *ELOVL6*, was found to be associated with variations in methylation patterns of the region between −529 bp and −350 bp and with *ELOVL6* expression. This region contains binding motifs for several regulators of the *ELOVL6* gene, which suggests that epigenetic changes may have a central role in the regulation of *ELOVL6* expression. Hence, we suggest that SNP *ELOVL6:c.-394G > A* is most likely responsible for the differential expression of *ELOVL6* and, consequently, for the palmitic and palmitoleic acid contents in muscle and backfat.

## Additional files

Additional file 1: Table S1. Title: Primers for the BAC screening (S) and the methylation study (M). Description: Table S1 shows all primers used for this work with the corresponding values of amplicon length (bp), melting temperature (Tm) and MgCl_2_ concentration. Primers used for the BAC screening are indicated with the letter “S” and primers for the methylation study with the letter “M”. Additional file 2: Table S2. Title: Association analyses for phenotypic data and the selected polymorphisms (*ELOVL6:c.-533C > T* and *ELOVL6:c.1922A > G*) and the haplotypes formed by the two SNPs. Description: Table S2 shows associations (shown with p-values) between *ELOVL6:c.-533C > T* (promoter) and *ELOVL6:c.1922A > G* (3’UTR); and between the haplotype formed by the two SNPs and backfat *ELOVL6* expression and C16:0 and C16:1(n-7) contents in IMF and backfat.
