# Supplementary material for: Epigenetic regulation of the ELOVL6 gene is associated with a major QTL effect on fatty acid composition in pigs
Source: Genet Sel Evol. 2015 Mar 25;47(1):20. doi: 10.1186/s12711-015-0111-y (PMC4371617; doi:10.1186/s12711-015-0111-y)
Supplement: Additional file 1: Table S1. — Title: Primers for the BAC screening (S) and the methylation study (M). Description: Table S1 shows all primers used for this work with the corresponding values of amplicon length (bp), melting temperature (Tm) and MgCl2 concentration. Primers used for the BAC screening are indicated with the letter “S” and primers for the methylation study with the letter “M”. [file 12711_2015_111_MOESM1_ESM.docx]

| **Name** | **Sequence (5’- 3’)*** | **Amplicon lenght (bp)** | **Tm** | **[MgCl_2_]** |
| --- | --- | --- | --- | --- |
| ELOVL6_P_Fw (S) | CCAGAGCTGGCAGGTTTTACTA | 605 | 60ºC | 2mM |
| ELOVL6_P_Rv (S) | CGGAGTCGCTACGTGTTCTCTA |  |  |  |
| ELOVL6_e2_Fw (S) | CCTGGTTTCTGCTCTGTATGCT | 94 | 60ºC | 2mM |
| ELOVL6_e2_Rv (S) | CAGCACTAATGGCTTCCTCAGTT |  |  |  |
| ELOVL6_e4_Fw (S) | TCACTGTGCTCCTGTACTCTTGG | 499 | 60ºC | 2mM |
| ELOVL6_e4_Rv (S) | TAAGCTGCCTTGGGTTTTGTG |  |  |  |
| Met_F1_Fw (M) | TGTGTTTTGTATTGGATTAGTTGG | 309 | 60ºC | 1.5mM |
| Met_F1_Rv (M) | [Btn]TCCCACRTAAAAAAATCAAACTTC |  |  |  |
| Met_Seq1 (M) | YGTTTTTAGTATTTTTAGATAT | ---- | ---- | ---- |
| Met_F2_Fw (M) | GATTTGGAGGGTGTGGTAAGAGTA | 232 | 60ºC | 1.5mM |
| Met_F2_Rv (M) | [Btn]TCATCCACAACCTCAATCCT |  |  |  |
| Met_Seq2 (M) | GAGGTGGGAAGTTTGA | ---- | ---- | ---- |
| Met_F3_Fw (M) | TAGGATTGAGGTTGTGGATGATT | 200 | 60ºC | 1.5mM |
| Met_F3_Rv (M) | [Btn]TCCATCACCCTTTTTACTTATCTACA |  |  |  |
| Met_Seq3 (M) | TTTTTTTYGTAAAGGGTTAAT | ---- | ---- | ---- |
| Met_F4_Fw (M) | TGATTTTTTTTTTTTGGTTATTAG | 183 | 58ºC | 2.5mM |
| Met_F4_Rv (M) | [Btn]AATCAAACTTCCCACCTCCTTAC |  |  |  |
| Met_Seq4 (M) | TGGTTATTAGTTATTTTTTATTTA | ---- | ---- | ---- |

**Additional file 1, Table S1:** Primers for the BAC screening (S) and the methylation study (M).

*Biotin-labelled primers are marked with [Btn].
