# Supplementary material for: Epigenetic regulation of the ELOVL6 gene is associated with a major QTL effect on fatty acid composition in pigs
Source: Genet Sel Evol. 2015 Mar 25;47(1):20. doi: 10.1186/s12711-015-0111-y (PMC4371617; doi:10.1186/s12711-015-0111-y)
Supplement: Additional file 2: Table S2. — Title: Association analyses for phenotypic data and the selected polymorphisms (ELOVL6:c.-533C > T and ELOVL6:c.1922A > G) and the haplotypes formed by the two SNPs. Description: Table S2 shows associations (shown with p-values) between ELOVL6:c.-533C > T (promoter) and ELOVL6:c.1922A > G (3’UTR); and between the haplotype formed by the two SNPs and backfat ELOVL6 expression and C16:0 and C16:1(n-7) contents in IMF and backfat. [file 12711_2015_111_MOESM2_ESM.docx]

| **Genetic**  **variants** | **Backfat gene expression** | **IMF** | | **Backfat** | |
| --- | --- | --- | --- | --- | --- |
|  |  | **C16:0** | **C16:1(n-7)** | **C16:0** | **C16:1(n-7)** |
| *ELOVL6:c.-533C>T* | 3.68x10^-03^ | 1.33x10^-03^ | 3.72x10^-04^ | 6.15x10^-10^ | 9.15x10^-04^ |
| *ELOVL6:c.1922A>G* | 4.46x10^-02^ | 6.96x10^-02^ | 6.98x10^-04^ | 5.44x10^-08^ | 3.57x10^-03^ |
| Haplotype | 7.22x10^-03^ | 1.81x10^-03^ | 6.68x10^-03^ | 1.42x10^-09^ | 9.66x10^-04^ |

**Additional file 2, Table S2:** Association analyses for phenotypic data and the selected polymorphisms (*ELOVL6:c.-533C>T* and *ELOVL6:c.1922A>G*) and the haplotype formed with both SNPs.
